# Gastrointestinal symptoms and association to food items in children with hirschsprung disease: a multinational patient-reported outcome study from Latin America and Spain

**DOI:** 10.1007/s00383-026-06605-1

**Published:** 2026-09-03

**Authors:** Carla Scaramella, Lovisa Telborn, Joseph Davidson, Hannah Day, Marta de Andres Crespo, Verónica Busoni, Pablo Lobos, Judith Lindert

**Affiliations:** 1 Asociación Marprung Tandil, Hirschsprung Argentina Coordinator, Reg. Charity N° 50825, 4 de Abril 1456, Tandil, Buenos Aires Argentina; 2 Department of Pediatric Surgery, Lund University, Skåne University Hospital, 22100 Lund, Sweden; 3 UCL GOSH Institute of Child Health, 30 Guilford Street, London, WC1N 1EH UK; 4 Department of Specialist Neonatal and Pediatric Surgery, Hannah Day, Great Ormond Street Hospital NHS Trust, London, WC1N 3JH UK; 5 Verónica Busoni - Chief of Pediatric Gastroenterology & Hepatology Service, Hospital Italiano de Buenos Aires, Tte. Juan Domingo Perón 4190, Ciudad de Buenos Aires, Argentina; 6 Pablo Lobos - Chief of Pediatric Surgery Service – Hospital Italiano de Buenos Aires, Tte. Juan Domingo Perón 4190, Ciudad de Buenos Aires, Argentina; 7 Department of Pediatric Surgery, University Medicine Rostock, Rostock , Germany

**Keywords:** Hirschsprung disease, Patient reported outcome, Bowel function, Diet and nutrition

## Abstract

**Purpose:**

Children with Hirschsprung disease (HD) frequently experience persistent bowel dysfunction after definitive surgery. Although many families report that diet influences gastrointestinal symptoms, evidence guiding dietary management remains limited. This study evaluated dietary habits, food-related gastrointestinal symptoms, and bowel function in children with HD across Spain and Latin America.

**Methods:**

A Spanish-language version of the OASIS Nutrition Questionnaire was developed by the multinational Holistic Care in Hirschsprung Disease Network and distributed through patient associations and social media. Children aged 6 months to 18 years with HD and their caregivers completed an anonymous online survey addressing demographics, disease characteristics, dietary habits, bowel function, and perceived food-related gastrointestinal symptoms. Functional outcomes were assessed using the Rintala Bowel Function Score in toilet-trained participants without an enterostomy.

**Results:**

A total of 108 responses from 11 Spanish-speaking countries were analysed. Disease extent included short-segment HD (39%), long-segment HD (36%), total colonic aganglionosis (16%), and small-bowel involvement (6.5%). Most participants (70%) followed a regular omnivorous diet.13% of participants used probiotics. Whereas 72% reported dietary exclusions and 66% had modified eating habits because of HD. Eating behaviors were affected in 66%, and 80% identified specific food items that worsened gastrointestinal symptoms. High-sugar foods (21%), dairy products (20%), pulses (12%), and fruits (10%) were the most frequently reported food items associated with adverse symptoms. The most common reported symptoms were soiling (51%) and abdominal bloating (29%). Patients with total colonic aganglionosis or small-bowel involvement reported more dietary gastrointestinal symptoms and the highest number of food-related symptoms.

**Conclusion:**

This first multinational Spanish speaking cohort survey demonstrates an association between food items and gastrointestinal symptoms in children with HD. Diet-related gastrointestinal symptoms are common in children with HD and increase with disease extent. Most families identified food items causing symptoms themselves. These findings highlight the need for individualized nutritional counselling as part of multidisciplinary long-term follow-up. Soiling was the main reported symptom in this Hispanic/Latino cohort. These findings emphasize the need for individualized dietary guidance supported by dietitian advice to support bowel function, growth, and quality of life, while preventing nutritional deficiencies.

## Introduction

Hirschsprung disease (HD) is a congenital disorder characterized by the absence of enteric ganglion cells in the distal bowel, resulting in functional intestinal obstruction. Definitive treatment consists of surgical resection of the aganglionic segment with pull-through of normally innervated bowel. Several established surgical techniques are available, with comparable long-term functional outcomes across approaches [1–7]. Despite adequate surgical treatment, many patients experience persistent bowel dysfunction and impaired continence. The most common long-term complications include constipation, fecal soiling, and Hirschsprung-associated enterocolitis [2–5]. These symptoms require ongoing management during long-term follow-up and may significantly impair quality of life (QoL) for patients and their families [2, 4, 5]. Dietary factors influencing well-being and bowel function are frequently reported by families of children with HD. Qualitative and survey-based studies indicate that parents perceive diet as an important determinant of gastrointestinal symptoms [3, 6]. This is supported by a recent multinational European patient-reported outcome survey including more than 560 children and adolescents with HD across seven countries [8]. In this cohort, over three-quarters of respondents reported that specific foods worsened gastrointestinal symptoms, and more than 60% indicated that HD had significantly altered eating behaviors and mealtime routines. Notably, 80% of families identified problematic foods independently, without guidance from healthcare professionals. Specific food groups have been associated with symptom exacerbation, most commonly pulses, high-sugar foods, fruits, and dairy products, whereas eggs, wheat, and soya were less frequently implicated [8]. Dietary effects were particularly prominent in patients with total colonic aganglionosis, with or without small-bowel involvement, who also reported greater social impairment related to bowel symptoms. These findings suggest heterogeneity in dietary responses and indicate that some symptoms traditionally attributed to postoperative bowel dysfunction may be diet-related and potentially modifiable. Adequate nutritional intake during childhood is essential for normal growth, neurodevelopment, immune function, and psychosocial well-being [9]. In children with HD, chronic bowel symptoms may disrupt daily activities, limit participation in school and social environments, and contribute to increased absenteeism and reduced academic performance [10]. Additionally, unsupervised dietary restrictions in response to perceived symptoms may increase the risk of nutritional deficiencies, particularly in children with more extensive disease [11, 12]. In children without HD presenting with chronic constipation, pharmacological therapy with stool softeners is generally recommended as first-line treatment [13]. Dietary interventions—including modification of fiber intake, increased fluid consumption, and reduction of cow’s milk—are commonly used in the management of pediatric functional constipation [14]. These dietary strategies are frequently extrapolated to children with Hirschsprung disease despite the absence of disease-specific evidence supporting their effectiveness in postoperative bowel dysfunction [15]. Current recommendations are therefore largely based on expert opinion and clinical experience rather than prospective interventional studies in HD. Although recent multinational survey data provide important insights into diet–symptom associations in HD, prospective studies evaluating targeted dietary interventions and their impact on bowel function and QoL remain limited.

Dietary habits differ substantially between geographical regions and cultures, potentially influencing both gastrointestinal symptoms and the perception of food-related symptoms. Most available patient-reported studies on nutrition in Hirschsprung disease have been conducted in Northern and Western Europe, where dietary patterns differ considerably from those in Latin America and Spain. Understanding whether food-related gastrointestinal symptoms differ across culturally diverse populations is important to develop culturally appropriate dietary recommendations for children with HD.

Improved understanding of food-related symptom patterns, derived from observational studies and patient-reported outcomes, may inform the design of future interventional studies. The present study uses patient-reported outcomes to evaluate the influence of diet on bowel function and gastrointestinal symptoms in children with HD, employing a validated Spanish translation of the original OASIS nutrition questionnaire.

## Methods

The questionnaire was based on the previously published OASIS Nutrition Questionnaire developed by the multinational OASIS (Holistic Care in Hirschsprung Disease) network to evaluate dietary habits, food-related gastrointestinal symptoms, bowel management and patient-reported outcomes in children with Hirschsprung disease. The original English questionnaire was developed by an international multidisciplinary panel including pediatric surgeons, pediatric gastroenterologists, dietitians and patient representatives [8]. The questionnaire should be considered an expert- and patient association-developed patient-reported survey instrument, as it had no formal psychometric validation. The Spanish version was produced by forward translation performed independently by medically trained native Spanish speakers, followed by consensus review within the multinational OASIS network to ensure conceptual equivalence.

The questionnaire was disseminated via national Hirschsprung Disease patient associations and social media across Spain and Central and South America. Children aged 6 months to 18 years with HD, together with their caregivers, were invited to participate. The questionnaire was open from January to November 2025. Participation was voluntary and anonymous without financial incentives. The full questionnaire is available as supplementary Material.

### Definitions and outcomes

Disease extent was classified according to the anatomical length of aganglionosis as short-segment Hirschsprung Disease (SSHD) when only the rectosigmoid was affected, long-segment Hirschsprung Disease(LSHD) proximal to the sigma but not involving the entire colon, total colonic aganglionosis (TCA), or colonic aganglionosis with small-bowel involvement. For analysis, patients were further grouped by the presence or absence of colon in situ (SSHD+LSHD vs. TCA ± small-bowel involvement).

A regular omnivorous diet was defined as an unrestricted diet including both plant- and animal-derived foods. Vegetarian, vegan, and pescatarian diets were classified separately.

High-sugar-containing foods were defined as: sweets, chocolate, or sugar-containing soda.

The survey assessed the perceived impact of specific food groups on bowel function using predefined symptom categories: changes in stool frequency, stool consistency, soiling, fecal incontinence, bloating, abdominal cramping, abdominal pain, flatulence, no symptoms, or other effects. Surveys were completed by parents or by patients themselves, depending on age and ability to respond, to capture patient-reported outcomes (PROs). Toilet-trained patients without an enterostomy were additionally asked to complete the Rintala Bowel Function Score (BFS; range 1–7). The Rintala Bowel Function classification is Score 1–20, 17–20 normal, 12–16 moderate, < 12 poor [1].

### Statistical analysis

Data were collected using REDCap and analyzed with SPSS version 26 (IBM). Results are presented as n (%) or median [interquartile range]. Group comparisons were performed using chi-square or Mann–Whitney U tests, as appropriate. Participants with incomplete responses were included if disease characteristics were available, resulting in variable denominators.

### Ethical approval

The study was conducted in accordance with the Helsinki Guidelines. Institutional Review Board approval was obtained by the coordinating center (University Medicine Rostock) from the Ethics Committee of the University of Rostock (July 2023; A 2023 − 0123). As only anonymous patient-reported questionnaire data were collected without intervention, additional local ethics approvals were not required in participating countries according to local regulations. The collaborating Partner Argentine ethics committee (Comité Independiente de Ética (CIE) para Ensayos en Farmacología Clínica Prof. Dr. Luis María Zieher’) reviewed the study and waived additional approval ‘.

## Results

### Characteristics of patients participating in the Spanish international questionnaire

**Fig. 1:**
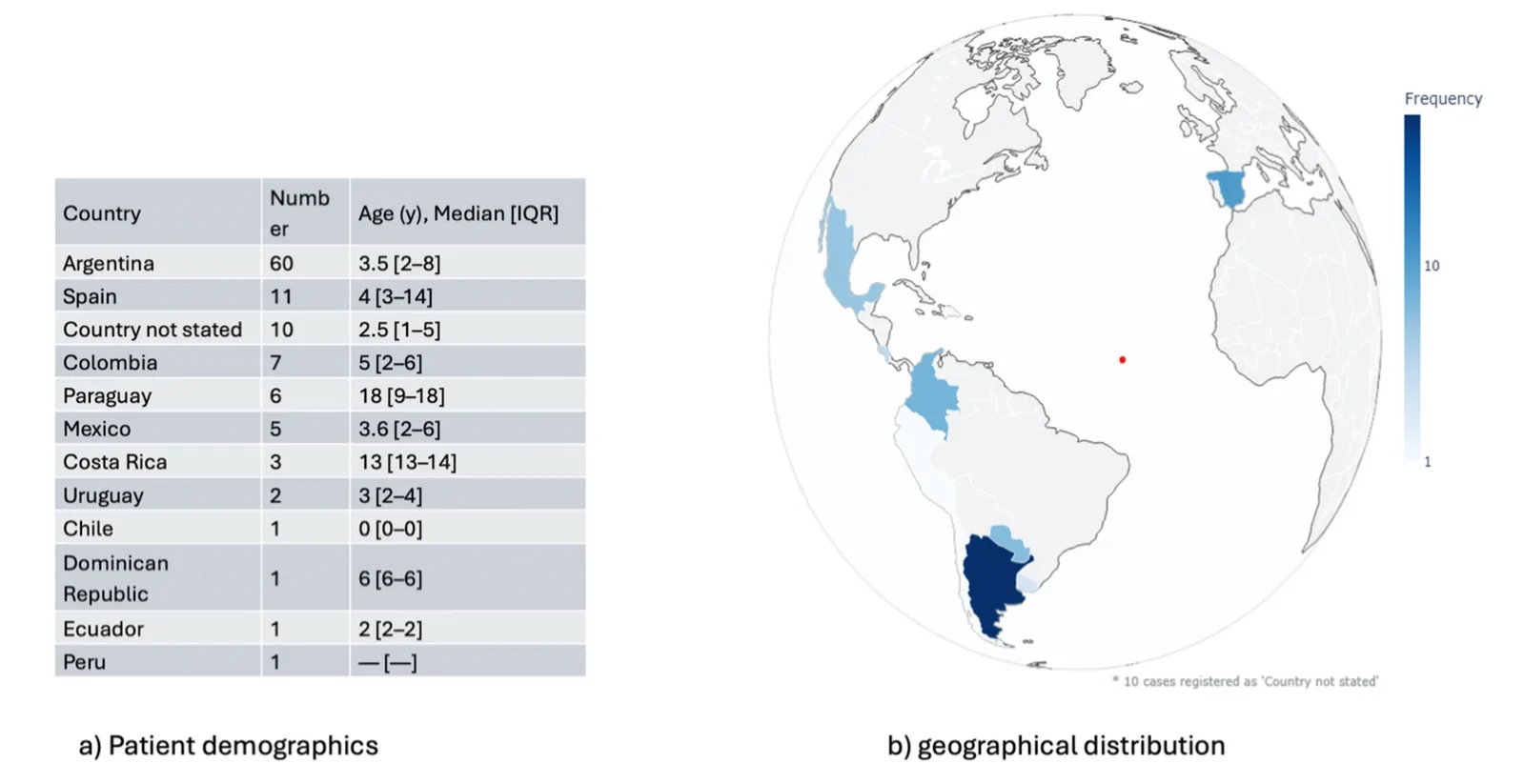
Geographical distribution and patient demographics

A total of 108 Spanish-speaking patients from 11 countries responded, including Argentina, Spain, Uruguay, Chile, Colombia, Paraguay, Mexico, Costa Rica, Ecuador, the Dominican Republic, and Peru (Fig. 1).

Short-segment disease accounted for 39% in the Overall cohort and (*n* = 42) of the cohort. Table 1.

**Table 1:** Level of disease

	Overall	Argentina	Spain	Rest of LATAM	Country not stated
Number	108	60	11	27	10
Age, y, median [IQR]	4.0 [2.0–8.0]	3.5 [2.0–7.25]	4.0 [2.5–11.0]	5.5 [2.0–9.0]	2.5 [1.0–5.0]
Segment, n (%)					
Rectosigmoid (short segment)	42 (38.9%)	30 (50.0%)	4 (36.4%)	5 (18.5%)	3 (30.0%)
Long segment	39 (36.1%)	18 (30.0%)	5 (45.5%)	12 (44.4%)	4 (40.0%)
Total colonic aganglionosis	17 (15.7%)	10 (16.7%)	1 (9.1%)	6 (22.2%)	0 (0.0%)
Small intestine involvement	7 (6.5%)	1 (1.7%)	1 (9.1%)	3 (11.1%)	2 (20.0%)
Not declared	3 (2.8%)	1	-	1	1
Current stoma (Yes/No) (%), n (%)	16/104 (15.4%)	6/59 (10.2%)	0/11 (0.0%)	7/25 (28.0%)	3/9 (33.3%)
Toilet-trained (without stoma), completed y/n	34/83 (42%)	21/51 (41.2%)	6/10 (60.0%)	6/16 (37.5%)	2/6 (33.3%)
ACE, n (%), completed y/n	7/104 (6.7%)	2/59 (3.4%)	0/11 (0.0%)	3/25 (12.0%)	2/9 (22.2%)
TAI, n (%), completed y/n	25/103 (24.3%)	12/59 (20.3%)	2/11 (18.2%)	9/24 (37.5%)	2/9 (22.2%)
Tube-feeding, n (%), completed y/n	2/103 (1.9%)	2/59 (3.4%)	0/11 (0.0%)	0/24 (0.0%)	0/9 (0.0%)

*ACE* Antegrade continence enema, *TAI* Transanal irrigation

*Denominator reflects the number of respondents with available data

### Bowel function in toilet-trained patients

Bowel function was assessed in toilet-trained patients without an enterostomy. Of the 104 patients without an enterostomy, *n* = 34 (33%) were toilet-trained. Only four patients aged ≥ 4 years had a stoma.

Table 2 presents patient-reported bowel function using the Rintala Bowel Function Scores (BFS) according to segment length. Functional outcomes did not differ significantly between the different disease extent groups. Across all HD segments, the median BFS was below 17. Considerable variability in scores was observed within anatomical subgroups. Patients with SSHD demonstrated a wide interquartile range (IQR 7–19), while LSHD also showed broad variability (range 3–20).

**Table 2:** Patient-reported bowel function according to the level of Hirschsprung disease

Item	Short disease (*n* = 9)	Long disease (*n* = 13)	TCA (*n* = 10)	Small intestine (*n* = 2)
Overall Score (Median [IQR])	9 [7–19]	12 [10–15]	14 [10–15]	8 [7–9, 11]
Are you aware of the feeling when you need to pass stool?				
Always aware	3 (33%)	5 (38%)	4 (40%)	1 (50%)
Most of the time	1 (11%)	3 (23%)	2 (20%)	0 (0%)
Often uncertain	2 (22%)	2 (15%)	1 (10%)	1 (50%)
No awareness	3 (33%)	3 (23%)	3 (30%)	0 (0%)
Are you able to hold back when you need to pass stools?				
Always able	4 (44%)	6 (46%)	6 (60%)	0 (0%)
Occasional problems holding in stool, less than once per week	0 (0%)	2 (15%)	1 (10%)	1 (50%)
Problems holding in stool every week	4 (44%)	3 (23%)	3 (30%)	0 (0%)
No control over bowels, problems every day	1 (11%)	2 (15%)	0 (0%)	1 (50%)
How often do you pass stool?				
Less than once every 2 days	1 (11%)	0 (0%)	0 (0%)	0 (0%)
Every 2 days	1 (11%)	2 (15%)	1 (10%)	0 (0%)
Once per day	4 (44%)	7 (54%)	0 (0%)	0 (0%)
Twice per day	2 (22%)	2 (15%)	2 (20%)	0 (0%)
More than twice per day	1 (11%)	2 (15%)	7 (70%)	2 (100%)
How often do you have issues with soiling or staining of the underwear?				
Never have issues with soiling	3 (33%)	2 (15%)	2 (20%)	0 (0%)
Soiling less than once a week, only rarely needing a change in underwear	0 (0%)	0 (0%)	0 (0%)	0 (0%)
Soiling every week, often requiring a change of underwear	3 (33%)	5 (38%)	4 (40%)	1 (50%)
Soiling all the time, using protective aids (i.e. pads or diapers)	1 (11%)	3 (23%)	0 (0%)	0 (0%)
How often do you have accidents with stools in the underwear?				
Never	4 (44%)	3 (23%)	2 (20%)	0 (0%)
Rarely, less than once per week	2 (22%)	5 (38%)	5 (50%)	1 (50%)
Weekly, wearing protective aids	3 (33%)	2 (15%)	1 (10%)	1 (50%)
Daily, wearing protective aids day and night	0 (0%)	3 (23%)	2 (20%)	0 (0%)
Do you suffer from constipation?				
No constipation at all	4 (44%)	5 (38%)	9 (90%)	1 (50%)
Constipation managed with diet	2 (22%)	4 (31%)	0 (0%)	0 (0%)
Constipation managed with medication	1 (11%)	1 (8%)	0 (0%)	0 (0%)
Constipation managed with enemas	2 (22%)	3 (23%)	1 (10%)	1 (50%)
What is the social impact of your bowel function?				
No impact on social life	3 (33%)	4 (31%)	4 (40%)	0 (0%)
Some impact (i.e. bad smells or need to get to toilet quickly)	2 (22%)	6 (46%)	3 (30%)	1 (50%)
Problems that limit social activities	1 (11%)	3 (23%)	1 (10%)	0 (0%)
Major social or psychological issues related to bowel function	3 (33%)	0 (0%)	2 (20%)	1 (50%)

The median Rintala BFS was similar across the different phenotypes of Hirschsprung Disease. The mean BFS in long-segment HD was 14 for TCA and 12 for LSHD.

Among school-aged children (≥ 6 years), continence rates were similar across HD segment groups. Continence was achieved in 7 of 8 patients with short-segment HD (87.5%), 9 of 12 with long-segment HD (75%), and 8 of 11 with TCA with or without small-bowel involvement (73%).

### Dietary characteristics in children already receiving complementary foods

#### Overall diet

70% of respondents (*n* = 75) reported following a regular omnivorous diet, including both plant- and animal-derived foods, while 2% (*n* = 4) followed vegetarian, vegan, or pescatarian diets. Specific dietary exclusions were reported by 72% (*n* = 78), most commonly involving dairy, gluten, FODMAP (Fermentable Oligosaccharides, Disaccharides, Monosaccharides and Polyols) -containing foods, or sugars.

Dietary fiber intake was monitored by 61% (*n* = 66) of respondents. Patients with short-segment disease more frequently reported higher fiber intake, whereas those with total colonic aganglionosis (TCA) tended to report lower fiber intake.

Dietary modifications attributed to HD were reported by 66% (*n* = 71), and 80% (*n* = 86) identified specific food triggers, most frequently among patients with TCA or small-bowel involvement (*p* < 0.001). Only 33% (*n* = 28) had discussed dietary changes with a healthcare professional (surgeon *n* = 18, general practitioner *n* = 20, dietitian *n* = 12), including 13 patients with TCA and 7 with small-bowel involvement.

Probiotic use was reported by 13% of participants (*n* = 14), 79% of patients with TCA or small-bowel involvement used probiotics. Approximately half of those specifying brands used multi-strain formulations (e.g., *Bacillus clausii*, *Bifidobacterium* spp., *Lactobacillus* spp.), while mono-strain preparations included *Saccharomyces boulardii* CNCM I-745 and *Limosilactobacillus reuteri* DSM 17,938.

#### Food-related dietary symptoms

Patients with TCA or small-bowel involvement reported the highest gastrointestinal symptom burden, with 55 gastrointestinal symptoms reported across 24 patients. Patients with LSHD reported 69 symptoms across 39 patients, while those with short-segment HD reported 50 symptom reports across 42 patients. There is a statistically significant difference in GI symptom burden between the three groups (Kruskal-Wallis H = 18.10, *p* = 0.0001). Post-hoc analysis reveals a significant difference between the Short Segment vs. TCA comparison (*p* = 0.000038): TCA patients report substantially higher GI symptom burden (mean = 31.59) compared to Short Segment patients (mean = 20.55).

The most frequently reported symptom was soiling/staining 50.9% (*n* = 55), followed by bloating or cramping 28.7% (*n* = 31).

**Fig. 2:**
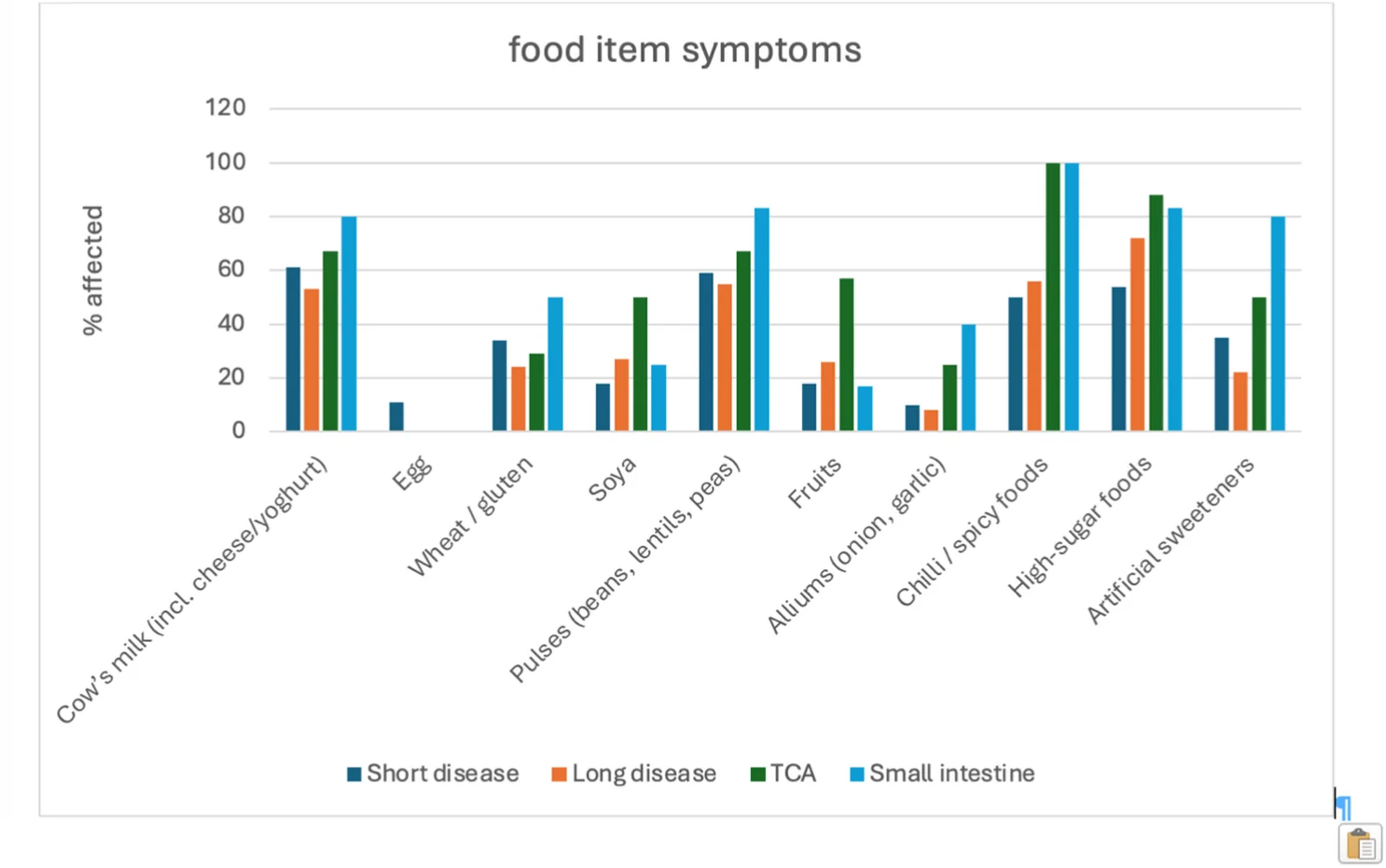
Food items causing gastrointestinal symptoms in association with level of disease

Foods most commonly associated with gastrointestinal symptoms included high-sugar foods, pulses, fruits, and dairy products. Lactose intolerance was reported in eight patients, and cow’s milk protein allergy in six. Wheat/gluten and artificial sweeteners were also identified as triggers; nine patients reported gluten intolerance, and one had confirmed coeliac disease. Eggs were rarely implicated but were reported to cause adverse abdominal symptoms in a small number of cases Fig. 2.

Some patients reported dermatological manifestations (*n* = 4), including dermatitis (*n* = 1), severe diaper rash (*n* = 1), or allergic reactions (*n* = 2) in our open-answer section. These were most frequently associated with cow’s milk, egg, gluten/wheat, pulses, and fruits, often correlating with documented allergies. The skin manifastion have not been a standard item of the questionnaire.

Symptom–food associations are detailed in Fig. 2 according to level of Disease. Pulses, High-Sugar Foods an Fruits are the three most reported food items associated with gastrointestinal symptoms. The reported symptoms are summarized in Table 3.

**Table 3:** Association of food items and bowel function

Food item (Number Affected)	Symptom (Top 3)	Prevalence of symptoms (%)
Pulses	Flatulence	60.5
	Bloating	41.9
	Cramping pains	34.9
High-sugar foods	More liquid stools	54.7
	Bloating	52.8
	Flatulence	45.3
Fruits	Increased stool frequency	60.0
	More liquid stools	60.0
	Bloating	48.0

Patients with rectosigmoid Disease report constipation in *n* = 9 (21.4%) and difficulty in evacuating stool. Soiling and Fecal Accidents are reported by 31% (*N* = 12) of patients with long-segment Hirschsprung disease, and Soiling and Fecal Incontinence are reported by 53% (*n* = 9) of patients with Total Colonic Aganglionosis. 71.4% (*n* = 5) of patients with small bowel involvement report liquid diarrhea-like output or liquid stools.

We could identify some regional patterns in the reported gastrointestinal symptoms.

#### Regional dietary patterns

Regional differences in food-related symptoms were observed. Patients from Argentina most frequently reported symptoms associated with cow’s milk and high-sugar foods, whereas pulse-related symptoms were most common in Spain 90%), followed by artificial sweeteners (40%). Respondents from the remaining Latin American countries more frequently reported symptoms following wheat/gluten (35%), pulses (50%), and chili/spicy foods (25%). Across all regions, cow’s milk was primarily associated with loose stools, bloating, abdominal cramping, flatulence, and soiling, while high-sugar foods were associated with loose stools, bloating, flatulence, stool accidents, and soiling. Pulses predominantly caused bloating and flatulence, fruits were mainly associated with loose stools and bloating, wheat/gluten with bloating and loose stools, and onion/garlic only occasionally caused symptoms.

## Discussion

This first multinational Spanish-language survey reports patient- and caregiver-reported outcomes from 108 participants across 11 Spanish-speaking countries, predominantly Argentina and Spain. Our findings demonstrate that food-related gastrointestinal symptoms are common in children with Hirschsprung disease (HD), with four out of five families identifying specific dietary items and two-thirds reporting dietary modifications due to Hirschsprung Disease. Patients with total colonic aganglionosis (TCA) or small bowel involvement reported a greater burden of food-related symptoms than those with short- or long-segment disease. These findings are consistent with the previously published European OASIS survey, suggesting that dietary concerns represent an important aspect of long-term management of Hirschsprung Disease across culturally diverse populations [8]. Similar to the European cohort, patients with extensive disease were overrepresented as participants, whereas short-segment HD accounted for only 39% of participants compared with the expected prevalence of 70–80% [8, 9]. This may reflect a degree of selection bias toward families with persistent bowel symptoms who might be more active in-patient associations [1, 11].

### Functional outcomes

Bowel function assessed using the Rintala Bowel Function Score (BFS) showed considerable variability across all HD subtypes [1, 10, 16]. Median BFS values did not differ significantly according to disease extent, indicating that long-term bowel function is influenced by factors beyond the length of aganglionosis, including surgical factors, bowel management, postoperative complications, and individual patient characteristics [2, 5, 11, 12, 14–16]. Although patients with TCA or small bowel involvement reported more diet-related symptoms, this was not accompanied by significantly poorer overall BFS, suggesting that bowel function and food-related symptom burden represent complementary but distinct outcome measures. Patients with SSHD had a huge variability in their reported bowel function score and also reported very low Bowel Function Score. Potentially, patients with impaired function are more likely to engage with self- help groups. Patients with SSHD reported fewer dietary concerns and less frequent probiotic use than those with more extensive disease, consistent with previous findings [8]. Current evidence does not support routine probiotic use after pull-through surgery, as systematic reviews have shown inconsistent benefit [17–21].

### Gastrointestinal symptoms

High-sugar foods, dairy products, pulses, and fruits were the most frequently reported dietary items associated with gastrointestinal symptoms. Soiling, bloating, and abdominal cramping were the main reported symptoms. These findings resemble those of the European OASIS survey despite differences in regional dietary habits [8]. Regional variations, including greater sensitivity to cow’s milk and high-sugar foods in Argentina and more pulse-related symptoms in Spain, could be influenced by differences in dietary exposure and cultural eating patterns rather than disease-specific mechanisms alone.

Despite the high prevalence of dietary symptoms, only one-third of families had discussed dietary modifications with a healthcare professional. Caregivers appear to develop dietary strategies independently, potentially having a risk of unnecessary dietary restrictions and nutritional deficiencies. Structured bowel management programmes and multidisciplinary follow-up should include specialist dietetic support and discuss dietary items associated with gastrointestinal symptoms to optimize long-term bowel function in children with HD [12, 14–16, 22].

In the open questions, some families reported skin rashes this gives the idea to systematically evaluate this further and also compare to the general paediatric population.

### Strengths and limitations

This is the first multinational Spanish-language study evaluating dietary habits and food-related gastrointestinal symptoms in children with HD across Spain and Latin America. Its strengths include a broad geographical representation and the use of a standardized patient-reported questionnaire, enabling direct comparison with the OASIS cohort [8].

Several limitations should be acknowledged. Recruitment through patient associations and social media may have introduced selection bias, reflected by the overrepresentation of patients with extensive disease and the underrepresentation of SSHD [8, 9]. Furthermore, the questionnaire has not undergone formal psychometric validation, although it was developed by the multinational OASIS collaborative as an expert and patient-representative collaboration and translated using a s forward-translation by native speakers. This analysis provides a subjective patient-reported view of bowel function and gastrointestinal symptoms in children with HD.

## Conclusion

This multinational patient-reported survey in a Spanish-speaking cohort with Hirschsprung Disease demonstrates that many children with Hirschsprung disease experience food-associated gastrointestinal symptoms, particularly after high-sugar foods, dairy products, pulses and fruits. Dietary concerns were more frequently reported in patients with more extensive disease. These findings support individualized dietary counselling within multidisciplinary long-term follow-up and further research on diet-related interventions in HD.

## Data Availability

No datasets were generated or analysed during the current study.

## Abbreviations

FODMAP: Fermentable oligosaccharides, disaccharides, monosaccharides and polyols
HD: Hirschsprung disease
LSHD: Long segment Hirschsprung disease
SSHD: Short segment Hirschsprung disease
QoL: Quality of life
TCA: Total colonic aganglionosis
